# Change in Lipofectamine Carrier as a Tool to Fine-Tune Immunostimulation of Nucleic Acid Nanoparticles

**DOI:** 10.3390/molecules28114484

**Published:** 2023-06-01

**Authors:** Hannah S. Newton, Yasmine Radwan, Jie Xu, Jeffrey D. Clogston, Marina A. Dobrovolskaia, Kirill A. Afonin

**Affiliations:** 1 Nanotechnology Characterization Laboratory, Cancer Research Technology Program, Frederick National Laboratory for Cancer Research Sponsored by the National Cancer Institute, Frederick, MD 21701, USA; hannah.newton@nih.gov (H.S.N.); jie.xu@nih.gov (J.X.); clogstonj@mail.nih.gov (J.D.C.); 2 Nanoscale Science Program, Department of Chemistry, University of North Carolina Charlotte, Charlotte, NC 28223, USA; yradwan@uncc.edu

**Keywords:** nucleic acid nanoparticles, lipofectamine, cytokine, interferons

## Abstract

Nucleic acid nanoparticles (NANPs) require a carrier to allow for their intracellular delivery to immune cells. Cytokine production, specifically type I and III interferons, allows for reliable monitoring of the carrier effect on NANP immunostimulation. Recent studies have shown that changes in the delivery platform (e.g., lipid-based carriers vs. dendrimers) can alter NANPs’ immunorecognition and downstream cytokine production in various immune cell populations. Herein, we used flow cytometry and measured cytokine induction to show how compositional variations in commercially available lipofectamine carriers impact the immunostimulatory properties of NANPs with different architectural characteristics.

## 1. Introduction

Nucleic acid nanoparticles (NANPs) are therapeutic nucleic acids designed to assemble into various geometric shapes with distinct physicochemical properties and have a host of diagnostic and therapeutic benefits in a wide array of diseases [1,2,3,4]. Physicochemical characterization and immunological evaluation of various RNA and DNA NANPs have been performed to fully understand their structure–activity relationship and help bridge gaps that hinder the clinical translation of these novel nanomaterials [3,5,6,7].

It has been shown that RNA and DNA NANPs require a carrier for their intracellular delivery to immune cells [5,6,7]. Without a delivery agent, NANPs have repetitively been shown to remain invisible to the immune system and do not stimulate immune responses [5,7,8]. However, upon delivery with, for example, Lipofectamine 2000 (L2K), NANPs are recognized by peripheral blood mononuclear cells (PBMCs), more so by monocytes than lymphocytes [5,7]. Furthermore, NANPs induce interferon (IFN) response in PBMCs, particularly type I (IFNα; IFNβ; IFNω) and III (IFNλ) IFN responses, and NANP composition and structure define the degree of response—for example, RNA NANPs stimulate greater immune response as compared to their DNA counterparts [5,6,7,8]. Within the RNA NANP category, the potency of IFN responses is influenced by nanoparticle architectures, shape, and size. For example, 3D RNA cubes are more immunostimulatory than 2D RNA rings, and 1D RNA fibers are the least immunostimulatory NANPs of all [5,8]; likewise, RNA hexagons are more potent than RNA triangles [5].

Moreover, various delivery platforms can tailor NANPs’ immunorecognition and subsequent function, including cytokine induction [7,8,9]. For example, NANPs’ delivery with dendrimers influences their uptake and PBMC cytokine induction when compared to L2K-assisted deliveries. NANPs delivered using cationic dendrimers induce pro-inflammatory cytokines and danger signals but not type I and III IFNs. In contrast, the same NANPs delivered with L2K induce the IFN response with no/low cytokines and danger signals [7]. To further examine the role of the delivery carrier in the qualitative and quantitative outcomes of NANPs’ interactions with the primary human immune cells, we investigate two different commercial lipofectamine carriers.

Lipofectamine is a 3:1 (*w*/*w*) formulation of 2,3-di-oleyloxy-N- [2(spermine-carboxamido)ethyl]-N,N-dimethyl-l-propan-aminium (DOSPA) and dioleoylphosphatidylethanolamine (DOPE) [10]. While L2K and Lipofectamine MessengerMAX (LMM) are both lipofectamines, their composition and chemical structures were optimized to improve the transfection of different types of nucleic acids. L2K is marketed as a more versatile transfection reagent with superior co-transfection performance and the ability to deliver a variety of nucleic acids [11]. LMM, on the other hand, is optimized and recommended for delivery of mRNA without genomic integration [12]. We hypothesized that fine structural variations in lipofectamine might further contribute to controlling the magnitude of NANP-mediated immunostimulation.

Herein, we present results indicating that the type of lipofectamine, L2K vs. LMM, alters NANPs’ immunostimulation and cytokine production, thereby providing additional tools to researchers for controlling the magnitude of the IFN response.

## 2. Results

### 2.1. Assembly of NANPs and Formation of Lipoplexes

**Figure 1:**
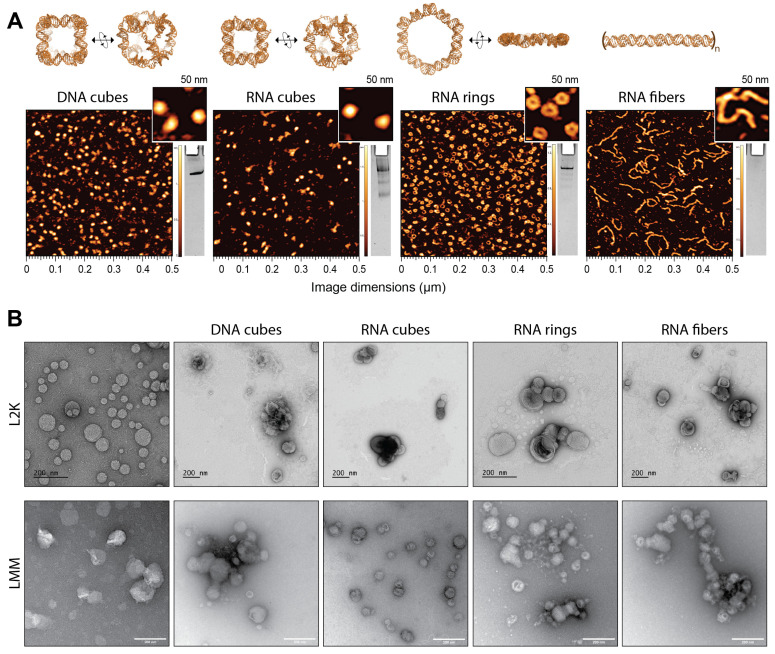
Characterization of NANPs and their lipoplexes. (**A**) 3D models and AFM images of representative NANPs. (**B**) TEM images of NANPs complexed with either L2K (upper panel) or LMM (lower panel).

A representative panel of NANPs—RNA fibers, RNA rings, RNA cubes, and DNA cubes—were selected to address the effect of NANPs’ composition and architectural parameters on their delivery with lipofectamines and immunorecognition. The assembly of NANPs took place in endotoxin-free conditions. The successful assembly of NANPs was confirmed using non-denaturing polyacrylamide gel electrophoresis (native-PAGE) and visualized via atomic force microscopy (AFM), as shown in Figure 1A.

In addition, eight lipoplexes formed between L2K or LMM, and each of the tested NANPs were visualized using transmission electron microscopy (TEM) and compared to free lipofectamines. The change in morphology of the carrier alone compared to the carriers complexed with NANPs suggests that NANPs were successfully complexed in L2K and LMM, as demonstrated in Figure 1B.

### 2.2. Monocytes Have Greater NANP Uptake Than Lymphocytes Regardless of Lipofectamine Carrier

**Figure 2:**
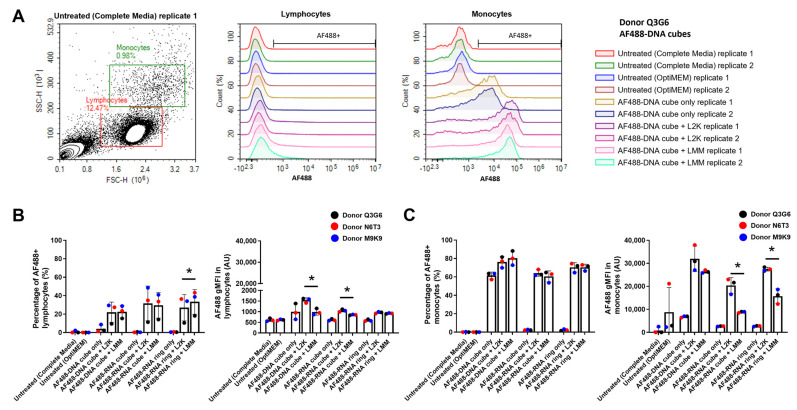
Monocytes have greater NANP uptake than lymphocytes regardless of lipofectamine carrier. PBMCs were treated with 10 nM AF488-NANPs for 20 h, fixed, and acquired on the flow cytometer. Cells were gated for lymphocyte and monocyte populations based on side and forward scatter and then gated on the AF488 signal. (**A**) Representative gating strategy for one healthy donor (Q3G6) showing the raw data for the negative controls and AF488-DNA cubes. (**B**) Lymphocytes and (**C**) monocytes were assessed for uptake of AF488-labeled NANPs. The percentage of cells positive for the AF488+ signal (left plots) and the geometric mean fluorescence intensity (gMFI) of AF488+ cells (right plots) were assessed for both populations. Each bar graph represents the mean data ± standard deviation from three healthy donors. Each dot represents the mean for each individual donor (run in duplicate). An asterisk (*) indicates *p* ≤ 0.05 for paired *t*-test between lipofectamine carriers for a particular NANP type. L2K—Lipofectamine^TM^ 2000 reagent; LMM—Lipofectamine^TM^ MessengerMAX^TM^ reagent.

To compare the ability of LMM vs. L2K to serve as carriers for NANPs, representative Alexa Fluor 488 (AF488) fluorescent DNA or RNA NANPs (AF488-DNA cubes; AF488-RNA cubes; and AF488-RNA rings) were incubated overnight with PBMCs at a final concentration of 10 nM. The uptake (and/or association with the cellular plasma membrane) of the fluorescent NANPs in both lymphocyte and monocyte populations was determined using flow cytometry. Lymphocyte and monocyte populations were defined via forward and side scatter. The NANP-association with the cells was measured in two ways: (i) the percentage of AF488+ lymphocytes or monocytes, i.e., the proportion of cells that have NANP-associated fluorescence, and (ii) the degree of geometric mean fluorescence intensity (gMFI) in each AF488+ population, i.e., the magnitude of NANP uptake/association by individual cells. Representative gating of the lymphocyte and monocyte populations, along with the AF488+ gating, is shown in Figure 2A.

As previously established by our group, AF488-labeled NANPs have different levels of fluorescence due to the differences in labeling efficiencies of individual oligos. Therefore, the experimental results should not be compared across different NANP types and should be considered qualitatively [5]. Nonetheless, our results were in agreement with previous data from our group, which showed lipofectamine leads to NANP uptake predominately by the monocyte population (Figure 2B,C) [5,7]. Both the percentage of AF488+ monocytes (~60–90%) and the gMFI of AF488+ monocytes (~10 K–40 K arbitrary units (a.u.)) were more significant than the results in the lymphocyte population (~10–50% and 800–1600 a.u., respectively), regardless of lipofectamine type (Figure 2B,C). However, there were a few significant differences when we compared L2K- versus LMM-mediated NANP uptake within a particular NANP type. The only difference between the percentage of AF488+ populations was in lymphocytes, where LMM led to a higher uptake percentage of AF488-RNA rings than L2K (Figure 2B). Furthermore, for the magnitude of NANP uptake, LMM led to lower gMFI for DNA and RNA cubes in the lymphocyte population, while LMM led to lower gMFI for RNA cubes and RNA rings in the monocyte population (Figure 2B,C). However, while these differences may be statistically significant, biological significance may not follow.

### 2.3. RNA Fibers Delivered with LMM Carrier Decrease IFN Production in PBMCs

**Figure 3:**
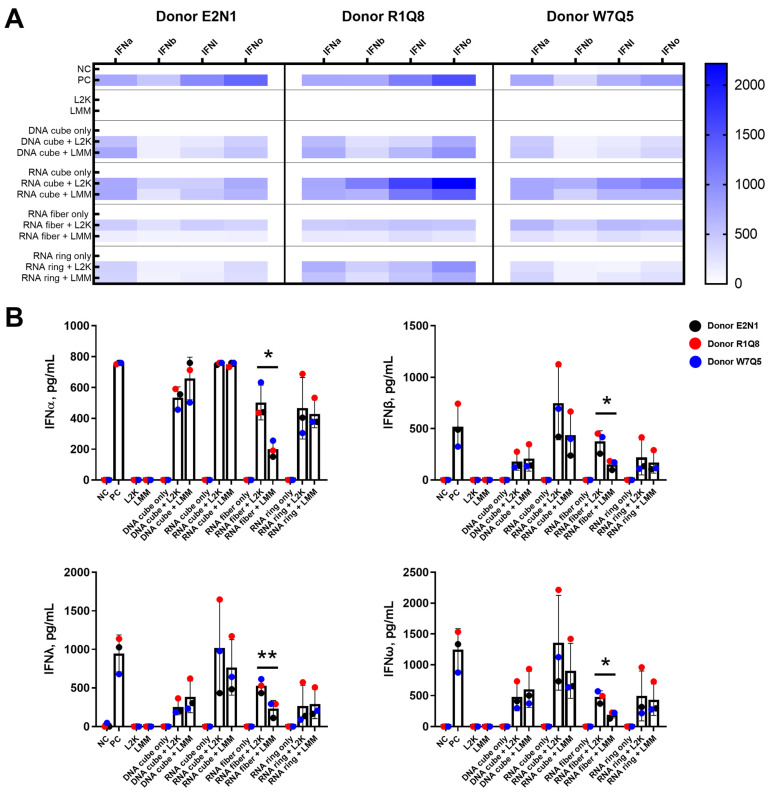
Lipofectamine carrier type alters PBMC IFN production in response to incubation with RNA fibers. PBMCs were treated with 10 nM NANPs for 20 h, and supernatants were collected and analyzed via multiplex for IFN production (IFNα; IFNβ; IFNλ; IFNω). (**A**) Heat map of the different IFN production levels of three healthy donors. Data points for each donor were run in duplicate. (**B**) Bar graphs representing the IFN production levels of three healthy donors. Each bar graph represents the mean data ± standard deviation from three healthy donors. Each dot represents the mean for each individual donor (run in duplicate). An asterisk (*) indicates *p* ≤ 0.05 or ** indicates *p* ≤ 0.01 for paired t-test between lipofectamine carriers for a particular NANP type. NC—negative control (untreated PBMC); PC—positive control (5 µg/mL ODN2216); L2K—Lipofectamine^TM^ 2000 reagent; LMM—Lipofectamine^TM^ MessengerMAX^TM^ reagent.

To determine if the lipofectamine-carrier-type-induced changes in NANP uptake affected PBMC biologically, IFN response was determined. Multiplex analysis was used to assess type I (IFNα; IFNβ; IFNω) and type III (IFNλ) interferon production in PBMCs after overnight treatment with 10 nM NANPs delivered using either L2K or LMM. The IFN panel was used in our earlier studies, which identified IFNs as biomarkers of immunostimulation of NANPs delivered using lipofectamine carriers [5,7]. The cytokine levels are presented as a heat map (Figure 3A) and as a bar graph (Figure 3B). It was determined that treatment with RNA cubes led to IFN levels similar to the positive control (ODN2216), and this finding agrees with previous studies (Figure 3) [5]. Furthermore, PBMC treatment with DNA rings, RNA fibers, and RNA rings generally led to lower IFN responses than the positive control.

When addressing the specific effect of lipofectamine carrier, we determined that lipofectamine carrier type did not affect NANP-induced IFN production except with the RNA fibers. In the case of RNA fibers, delivery of RNA fibers with LMM led to decreased IFN production for all four IFNs tested as compared to L2K (Figure 3B). This difference in IFN production may reflect the design of the LMM carrier, which was optimized to deliver mRNA.

## 3. Discussion

The greater degree of NANP uptake in the monocyte population as compared to the lymphocyte population in the presence of a lipofectamine carrier (Figure 2) is consistent with our previous studies [5,7] and data published by other research groups using DNA origami [13]. The uptake of these NANPs in the monocytes was higher than in lymphocytes regardless of tested carriers—L2K, LMM (Figure 2), or dendrimers [7]. Furthermore, while there were differences seen between L2K-mediated and LMM-mediated uptake for a few NANPs in the lymphocyte and monocyte populations, the differences are less than two-fold except for AF488 gMFI in monocytes for RNA cubes (Figure 2B,C). Differences less than two-fold are unlikely to lead to a biologically significant change. Moreover, these differences seen in uptake did not correspond to downstream differences in IFN production (Figure 3). We observed decreased PBMC IFN production after treatment with LMM-delivered RNA fibers compared to L2K-delivered RNA fibers. Unfortunately, we did not have AF488-RNA fibers to test RNA fiber uptake in monocyte and lymphocyte populations. Therefore, we do not have data to indicate whether the decrease in IFN production in PBMC from LMM-delivered RNA fibers is due to a lack of NANP uptake or another downstream process.

Interestingly, the current study and one of our earlier studies [7] observed the association of DNA cubes with ~60% of the monocyte population in the absence of a carrier (Figure 2C, left) [7]. This observation was also similar to the study by Du et al. investigating the uptake of DNA origami [13] but in contrast to the initial report by Hong et al., in which the uptake of DNA NANPs by monocytes was detected only in the presence of L2K [5]. We hypothesize that differences in the type of flow cytometer used in these studies may explain the observed discrepancy in the test results. Our current research and reports by Avila et al. and Du et al. utilized digital flow cytometers, which adjust the instrument settings automatically and, thus, are more sensitive at detecting even low fluorescent signal [7,13]. In contrast, the initial study by Hong et al. used a traditional cytometer which involves manual adjustment of instrument settings and often leads to the relocation of objects with weak fluorescence outside of the data collection gates [5]. Furthermore, even though the use of the NovoCyte flow cytometer in our current study revealed ~60% of the monocyte population was positive for DNA cube in the absence of any carrier (Figure 2C, left), this increase was not accompanied by increased AF488 gMFI (Figure 2C, right) nor was it accompanied by detectable IFN production (Figure 3), further suggesting that the association on the individual cell level was relatively low. This could imply that the NovoCyte 3005 (and possibly other digital cytometers with similar properties) is more sensitive than the previously used FACSCalibur [5] in the ability to detect low NANP quantities associated with the cells. A cross-validation between the two instruments would help verify this hypothesis, but it was not feasible because FACSCalibur is no longer available.

## 4. Materials and Methods

### 4.1. Materials

DNA strands (PCR forward and reverse primers and templates for RNA NANPs and individual oligos for DNA NANPs) and fluorescently labeled oligos (3′- Alexa Fluor 488) were obtained from Integrated DNA Technologies (IDT), Inc. MyTaq Mix, was purchased from Bioline. A DNA Clean & Concentrator kit was obtained from Zymo Research. RQ1 RNase-Free DNase was purchased from Promega (3 u/50 µL). Phosphate-buffered saline (PBS), RPMI-1640 medium, penicillin–streptomycin solution, L-glutamine, ficoll-paque premium, fetal bovine serum (FBS), and HyPure cell-culture-grade water were all obtained from Cytiva/GE Heathcare Life Sciences (Marlborough, MA, USA). Opti-MEM^TM^ I reduced serum medium and Hank’s balanced salt solution (HBSS) were from Gibco (Gaithersburg, MD, USA). Acridine orange (AO)/propidium iodide (PI) staining solution was from Nexcelom Bioscience (Lawrence, MA, USA). Oligodeoxyribonucleotide, a human TLR9 ligand (ODN2216), was from InvivoGen (San Diego, CA, USA). NovoFlow, NovoRinse, and NovoClean were from Agilent Technologies (Santa Clara, CA, USA). Lipofectamine^TM^ MessengerMAX^TM^ reagent and Lipofectamine^TM^ 2000 reagent were obtained from Invitrogen (Waltham, MA, USA). Paraformaldehyde (PFA) 20% Solution was from Electron Microscopy Science (Hatfield, PA, USA). A custom 4-plex Multiplex (IFNα; IFNβ; IFNλ; IFNω) kit with sample diluent, calibrator 1, calibrator 2, detection solution, streptavidin-HRP, substrate A, substrate B+, and wash buffer was obtained from Quansys BioSciences (Logan, UT, USA).

### 4.2. NANP Preparation

All sequences used for NANP preparation are provided in the Supporting Information. DNA templates were amplified via PCR using MyTaq Mix. The DNA Clean & Concentrator kit was used to purify the amplified PCR products, followed by in vitro run-off transcription using T7 RNA Polymerase in 80 mM HEPES-KOH (pH 7.5), 2.5 mM spermidine, 50 mM DTT, 25 mM MgCl_2_, and 5 mM of each rNTP at 37 °C over 3.5 h. Transcription was stopped through adding RQ1 RNase-Free DNase and incubating at 37 °C for 30 min. For the purification of RNA strands, denaturing polyacrylamide gel electrophoresis (PAGE, 8%) in the presence of 8 m urea run in 89 mM tris-borate, 2 mM EDTA (TBE, pH 8.2) was run at 13 W for 2 h. UV was used to visualize the RNA bands; the bands were then excised and eluted overnight in 300 mM NaCl, TBE (pH 8.2) at 4 °C. To precipitate the RNAs, the elution was mixed with 2.5 volumes of 100% EtOH and stored at −20 °C for 3 h. Then, the samples were centrifuged at 10.0× *g* for 30 min at 4 °C. The pellet was washed with 90% EtOH for 10 min via centrifugation at 10.0× *g* at 4 °C; this step was repeated twice. The pelleted samples were vacuum-dried at 55 °C with IR in a CentriVap micro-IR vacuum concentrator (Labconco), then dissolved in HyPure cell-culture-grade water. The concentration of each strand was measured using a NanoDrop 2000 (ThermoFisher) at 260 nm. The fourteen RNA strands were stored at −20 °C until use.

All NANPs were assembled in a one-pot thermal anneal through combining each strand in an equimolar ratio with HyPure cell-culture-grade water. The DNA cubes, RNA cubes, AF488-DNA cubes, and AF488-RNA cubes were heated to 95 °C for 2 min, then mixed with assembly buffer (89 mM tris-borate (pH 8.2), 2 mM MgCl_2_, 50 mM KCl) and incubated at 45 °C for 30 min, and then stored at 4 °C until use. The RNA rings, AF488-RNA fibers, and RNA rings were heated to 95 °C for 2 min, snap-cooled on ice for 2 min, mixed with the assembly buffer, and incubated at 30 °C for 30 min, then stored at 4 °C until use.

### 4.3. Characterization of NANPs

Successful assembly of NANPs was confirmed via visualization on 8% native-PAGE (37.5:1 acrylamide:bis-acrylamide). The gel was prepared on a Mini-PROTEAN Tetra Cell system (Bio-Rad), pre-run for 5 min at 150 V with running buffer (89 mM TB (pH 8.2), and 2 mM MgCl_2_). 2 µL of each sample was mixed with 2 µL loading buffer (Assembly buffer, 30% glycerol, bromophenol blue, xylene cyanol), and loaded per well. The loaded gel was run at 300 V for 30 min in a 4 °C cold room. The gel was stained with ethidium bromide (EtBr, 0.5 µg mL^−1^) for 5 min, then washed twice with double-deionized water (ddiH2O). Then, the gel was imaged using a ChemiDoc MP (Bio-Rad). The Alexa Fluor 488-labeled NANPs’ gel was imaged before EtBr staining via the Alexa Fluor 488 setting on the ChemiDoc MP system.

Atomic force microscopy (AFM) imaging of NANPs was performed on a freshly cleaved 1-(3-aminopropyl) silatrane-modified mica surface as previously described [7,14,15]. The AFM imaging was performed in tapping mode on the MultiMode AFM Nanoscope IV system (Bruker Instruments, Billerica, MA, USA).

For TEM imaging, 10 µL of corresponding 1 µM NANP stock and 2 µL of L2K or LMM were repeatedly mixed through pipetting up and down. The complexed samples were incubated at room temperature for 5–30 min. Stock L2K and LMM complexes were used for imaging except for the LMM + RNA fiber, which was diluted 10-fold in water before imaging. Samples were vortexed and 5 µL of each sample was applied to a glow-discharged carbon-coated 200 mesh Cu grid (EMS, Hatfield, PA, USA) for LMM/LMM-NANPs complexes or carbon-coated 400 mesh Cu/Rh grid (Ted Pella, Redding, CA, USA) for L2K/L2K-NANPs complexes and allowed to dry for 1 min at room temperature. Staining with 5 µL of 1% uranyl acetate (EMS, Hatfield, PA for LMM samples or Polysciences, Warrington, PA, USA for L2K samples) was repeated twice followed by final blotting and air-drying the grid. An FEI Tecnai T20 transmission electron microscope operating at 200 kV with a Gatan 2 k × 2 k Eagle camera was used to image the LMM grids and an FEI Talos L120C TEM with Gatan 4 k × 4 k OneView camera was used to image the L2K grids. A bridging experiment analyzing L2K on 200 mesh Cu grids (EMS, Hatfield, PA, USA) and images from an FEI Tecnai T20 transmission electron microscope operating at 200 kV with a Gatan 2 k × 2 k Eagle camera was conducted to verify that differences in instrumentation do not affect the results; the image is included in the Supplementary Materials (Figure S1).

### 4.4. PBMC Isolation

Healthy human donor whole blood was collected in li-heparin vacutainers (BD BioSciences) under NCI-Frederick protocol OH9-C-N046. The whole blood was used for PBMC isolation as specified in NCL protocol ITA-10 [16]. In brief, whole blood was diluted with PBS at a 1:1 ratio, layered over ficoll-paque at a ratio of 4:3 (4 mL diluted blood for every 3 mL ficoll-paque), and centrifuged for 30 min at room temperature at 900× *g* with no brake. The mononuclear cell layer containing the PBMCs was then removed, collected, and washed twice with HBSS (centrifuged for 10 min at 400× *g*). The PBMCs were resuspended in complete RPMI-1640 medium (10% heat-inactivated FBS, 100 U/mL penicillin, 100 µg/mL streptomycin, and 2 mM L-glutamine) and counted on a Cellometer using a 1:1 ratio of the cell suspension to AOPI. Once the PBMCs were counted, samples were diluted to 1.25 × 10^6^ cells/mL using a complete RPMI-1640 medium.

### 4.5. Uptake of Alexa Fluor-488 NANPs in PBMCs

PBMCs were aliquoted into a 96-well round bottom plate with 160 µL cell suspension (1.25 × 10^6^ cells/mL) per well. The AlexaFluor-488 NANPs (AF488-DNA cubes; AF488-RNA cubes; and AF488-RNA rings) and appropriate controls (untreated controls and no-carrier controls) were then prepared in microcentrifuge tubes using Opti-MEM^TM^ I reduced serum medium and lipofectamine reagents. An aliquot of 15 µL of 1 µM stock of appropriate NANPs was combined with 3 µL of lipofectamine reagent (LMM or L2K) or 3 µL of Opti-MEM^TM^ I reduced serum medium for the no-carrier controls and incubated between 5–30 min in the dark at room temperature. The untreated controls consisted of either complete RPMI-1640 media (Complete Media) only or Opti-MEM^TM^ I reduced serum medium (OptiMEM) only. After the incubation, 282 µL of Opti-MEM^TM^ I reduced serum medium was added to each sample (except negative controls) for a total volume of 300 µL and an NANP concentration of 50 nM where applicable. An aliquot of 40 µL prepared sample or control was added to each appropriate well of the prepared 96-well plate with PBMC suspension for a final volume of 200 µL (cells at 1 × 10^6^ cells/mL; NANP at 10 nM final concentration). The 96-well plate was placed in a humidified 37 °C/95% CO_2_ incubator for approximately 20 h.

The PBMC samples were then prepared for acquisition on a NovoCyte 3005 flow cytometer (Agilent Technologies, Inc., Santa Clara, CA, USA). The plate was removed from the incubator and centrifuged for 5 min at 400× *g*. The supernatants from each well were then aspirated and discarded, leaving the cell pellet undisturbed. The samples were washed twice with 150 µL 1× PBS (centrifuged 400× *g* for 5 min). The cell pellets were then fixed with 2% PFA for 15 min at room temperature and washed twice more with 1× PBS. Each cell pellet was resuspended in 150 µL 1× PBS for acquisition on the flow cytometer. On the NovoExpress software, side-scatter and forward-scatter area and height parameters were selected along with the area and height parameters for the FITC (488) channel. All other parameters remained unselected. Samples were then acquired with the instrumentation and analyzed using GraphPad Prism 9 (Graph Pad Software, Boston, MA, USA) and NovoExpress software version 1.5.6 (Agilent Technologies, Inc., Santa Clara, CA, USA).

### 4.6. IFN Production of PBMCs after NANPs Treatment

PBMCs were aliquoted into 96-well round bottom plates with 160 µL cell suspension (1.25 × 10^6^ cells/mL) per well. The NANPs (DNA cubes; RNA cubes; RNA fibers; and RNA rings) and appropriate controls (negative control, positive control (5 µg/mL ODN2216), vehicle controls, no-carrier controls) were then prepared in microcentrifuge tubes. An aliquot of 20 µL of 1 µM stock of appropriate NANPs was combined with 4 µL of lipofectamine reagent (LMM or L2K) or 4 µL of Opti-MEM^TM^ I reduced serum medium for the no-carrier controls and incubated between 5–30 min at room temperature. The vehicle controls consisted of 20 µL Opti-MEM^TM^ I reduced serum medium combined with 4 µL of appropriate lipofectamine reagent. The negative control consisted of Opti-MEM^TM^ I decreased serum medium only. The positive control consisted of 10 µL ODN2216 1 mg/mL stock diluted in 390 µL Opti-MEM^TM^ I reduced serum medium for a 25 µg/mL concentration. After the incubation, 376 µL of Opti-MEM^TM^ I reduced serum medium was added to each sample (except the positive control) for a total volume of 400 µL and a NANPs concentration of 50 nM where applicable. An aliquot of 40 µL prepared sample or control was added to each appropriate well of the prepared 96-well plates with PBMC suspension for a final volume of 200 µL (cells at 1 × 10^6^ cell/mL). NANP samples were at a 10 nM final concentration, and the positive control samples were at a final concentration of 5 µg/mL. The well plates were placed in a humidified 37 °C/95% CO_2_ incubator for approximately 20 h. After the incubation, the plates were centrifuged for 10 min at 700× *g*. Supernatant aliquots were then collected in newly labeled 96-well plates and stored at −80 °C.

A custom 4-plex Multiplex (IFNα; IFNβ; IFNλ; IFNω) from Quansys BioSciences was then used to analyze the freeze–thawed aliquots according to the manufacturer’s manual and NCL Protocol ITA-27 [17]. All reagents needed were included in the kit except for de-ionized water and cell-culture-grade water and prepared when indicated by the manual. In brief, the supernatants were thawed (partially at room temperature and partially at 37 °C). The calibration standards were prepared using the sample diluent in a 96-well polypropylene plate. The supernatant samples were diluted 2-fold with the sample diluent. 50 µL aliquots of calibration standards and supernatants were loaded into appropriate wells of the provided multiplex plate and incubated at room temperature for 2 h on a shaker (500 rpm). The multiplex plate was washed 3 times with wash buffer using a plate washer. The detection mix was then added to the multiplex plate and incubated for 1 h at room temperature on a shaker. The multiplex plate was then washed 3 times. A 50 µL aliquot of streptavidin-HRP was added to each well, and the plate was incubated for 20 min on the shaker. The multiplex plate was then washed 6 times, and 50 µL of ChemiLum substrate (Substrate A combined with Substrate B+) was added to each well. The plate was then read using the Quansys ImagePro, and the resulting data were analyzed using Microsoft Excel and GraphPad Prism.

## 5. Conclusions

This is the first study to demonstrate that the lipofectamine type of commercial delivery agents can be used as a simple tool to mediate change in the immunorecognition of different NANPs. LMM decreased IFN production in response to RNA fibers, which may be linked to their linear structure. This sensitivity to lipofectamine carriers could be used to modify PBMC response to NANPs precisely.

## Data Availability

All data are provided in manuscript.

## Supplementary Materials

The following supporting information can be downloaded at: https://www.mdpi.com/article/10.3390/molecules28114484/s1, Sequences used in this project and Figure S1: Comparison of lipofectamine 2000 (L2K) morphology by TEM performed using different grids.

## Sample Availability

Samples of the compounds (NANPs) are available from the authors upon reasonable request.
