# Supplementary material for: Change in Lipofectamine Carrier as a Tool to Fine-Tune Immunostimulation of Nucleic Acid Nanoparticles
Source: Molecules. 2023 Jun 1;28(11):4484. doi: 10.3390/molecules28114484 (PMC10254523; doi:10.3390/molecules28114484)
Supplement: Supplementary file 1 [file molecules-28-04484-s001.zip › molecules-2396823-supplementary.pdf]

Supplementary Information

Title:

Change in lipofectamine carrier as a tool to fine-tune immunostimulation of nucleic acid nanoparticles

Hannah S. Newton[1,+], Yasmine Radwan[2,+], Jie Xu[1], Jeffrey D. Clogston[1], Marina A. Dobrovolskaia[1,*] and Kirill A. Afonin[2,*]

1 Nanotechnology Characterization Laboratory, Cancer Research Technology Program, Frederick National Laboratory for Cancer Research sponsored by the National Cancer Institute, Frederick, MD 21701, USA

2 Nanoscale Science Program, Department of Chemistry, University of North Carolina Charlotte, Charlotte, NC 28223, USA

[+] - Authors contributed equally

* - Correspondence: MAD: marina@mail.nih.gov; KAA: kafonin@uncc.edu

Sequences used in this project

DNA Cubes

	Sequence	#NTs	MW (g/mole)
5'	GGCAACTTTGATCCCTCGGTTTAGCGCCGGCCTTTTCTCCCACACTTTCACG	52	15784.2
5'	GGGAAATTTCGTGGTAGGTTTTGTTGCCCGTGTTTCTACGATTACTTTGGTC	52	16074.4
5'	GGACATTTTCGAGACAGCATTTTTTCCCGACCTTTGCGGATTGTATTTTAGG	52	15996.4
5'	GGCGCTTTTGACCTTCTGCTTTATGTCCCCTATTTCTTAATGACTTTTGGCC	52	15825.2
5'	GGGAGATTTAGTCATTAAGTTTTACAATCCGCTTTGTAATCGTAGTTTGTGT	52	16074.5
5'	GGGATCTTTACCTACCACGTTTTGCTGTCTCGTTTGCAGAAGGTCTTTCCGA	52	15933.3

RNA Cubes

	Sequence	#NTs	MW (g/mole)
5'	GGCAACUUUGAUCCCUCGGUUUAGCGCCGGCCUUUUCUCCCACACUUUCACG	52	16391.7
5'	GGGAAAUUUCGUGGUAGGUUUUGUUGCCCGUGUUUCUACGAUUACUUUGGUC	52	16597.8
5'	GGACAUUUUCGAGACAGCAUUUUUUCCCGACCUUUGCGGAUUGUAUUUUAGG	52	16547.8
5'	GGCGCUUUUGACCUUCUGCUUUAUGUCCCCUAUUUCUUAAUGACUUUUGGCC	52	16334.6
5'	GGGAGAUUUAGUCAUUAAGUUUUACAAUCCGCUUUGUAAUCGUAGUUUGUGU	52	16597.8
5'	GGGAUCUUUACCUACCACGUUUUGCUGUCUCGUUUGCAGAAGGUCUUUCCGA	52	16498.8

RNA Rings

	Sequence	#NTs	MW (g/mole)
5'	GGGAACCGUCCACUGGUUCCCGCUACGAGAGCCUGCCUCGUAGC	44	14062.5
5'	GGGAACCGCAGGCUGGUUCCCGCUACGAGAGAACGCCUCGUAGC	44	14188.6
5'	GGGAACCGCGUUCUGGUUCCCGCUACGAGACGUCUCCUCGUAGC	44	14040.4
5'	GGGAACCGAGACGUGGUUCCCGCUACGAGUCGUGGUCUCGUAGC	44	14183.5
5'	GGGAACCACCACGAGGUUCCCGCUACGAGAACCAUCCUCGUAGC	44	14076.5
5'	GGGAACCGAUGGUUGGUUCCCGCUACGAGAGUGGACCUCGUAGC	44	14207.5

RNA Fibers

	Sequence	Strand	#NTs	MW (g/mole)
5'	GGGAAUCCAAGGAGGCAGGAUUCCCGUCACAGAAGGAGGCACUGUGAC	1	48	15609.5
5'	GGGAACGUAAGCCUCCAACGUUCCCGGAUGCUAAGCCUCCAAGCAUCC	HIV 2	48	15323.3

AF488-DNA cubes

	Sequence	#NTs	MW
5'	GGCAACTTTGATCCCTCGGTTTAGCGCCGGCCTTTTCTCCCACACTTTCACG	52	15784.2
5'	GGGAAATTTCGTGGTAGGTTTTGTTGCCCGTGTTTCTACGATTACTTTGGTC	52	16074.4
5'	GGACATTTTCGAGACAGCATTTTTTCCCGACCTTTGCGGATTGTATTTTAGG	52	15996.4
5'	GGCGCTTTTGACCTTCTGCTTTATGTCCCCTATTTCTTAATGACTTTTGGCC /3AlexF488N/-3'	52	16552.9
5'	GGGAGATTTAGTCATTAAGTTTTACAATCCGCTTTGTAATCGTAGTTTGTGT	52	16074.5
5'	GGGATCTTTACCTACCACGTTTTGCTGTCTCGTTTGCAGAAGGTCTTTCCGA	52	15933.3

AF488-RNA cubes

	Sequence	#NTs	MW (g/mole)
5'	GGCAACUUUGAUCCCUCGGUUUAGCGCCGGCCUUUUCUCCCACACUUUCACG	52	16391.7
5'	GGGAAAUUUCGUGGUAGGUUUUGUUGCCCGUGUUUCUACGAUUACUUUGGUC	52	16597.8
5'	GGACAUUUUCGAGACAGCAUUUUUUCCCGACCUUUGCGGAUUGUAUUUUAGG	52	16547.8
5'	GGCGCUUUUGACCUUCUGCUUUAUGUCCCCUAUUUCUUAAUGACUUUUGGCC /3AlexF488N/-3'	52	17062.2
5'	GGGAGAUUUAGUCAUUAAGUUUUACAAUCCGCUUUGUAAUCGUAGUUUGUGU	52	16597.8
5'	GGGAUCUUUACCUACCACGUUUUGCUGUCUCGUUUGCAGAAGGUCUUUCCGA	52	16498.8

AF488-RNA rings

	SEQUENCE	#NTs	MW (g/mole)
5'	GGGAACCGUCCACUGGUUCCCGCUACGAGAGCCUGCCUCGUAGC	44	14062.5
5'	GGGAACCGCAGGCUGGUUCCCGCUACGAGAGAACGCCUCGUAGC	44	14188.6
5'	GGGAACCGCGUUCUGGUUCCCGCUACGAGACGUCUCCUCGUAGC/3AlexF488N/-3'	44	14768.0

5'	GGGAACCGAGACGUGGUUCCCGCUACGAGUCGUGGUCUCGUAGC	44	14183.5
5'	GGGAACCACCACGAGGUUCCCGCUACGAGAACCAUCCUCGUAGC	44	14076.5
5'	GGGAACCGAUGGUUGGUUCCCGCUACGAGAGUGGACCUCGUAGC	44	14207.5

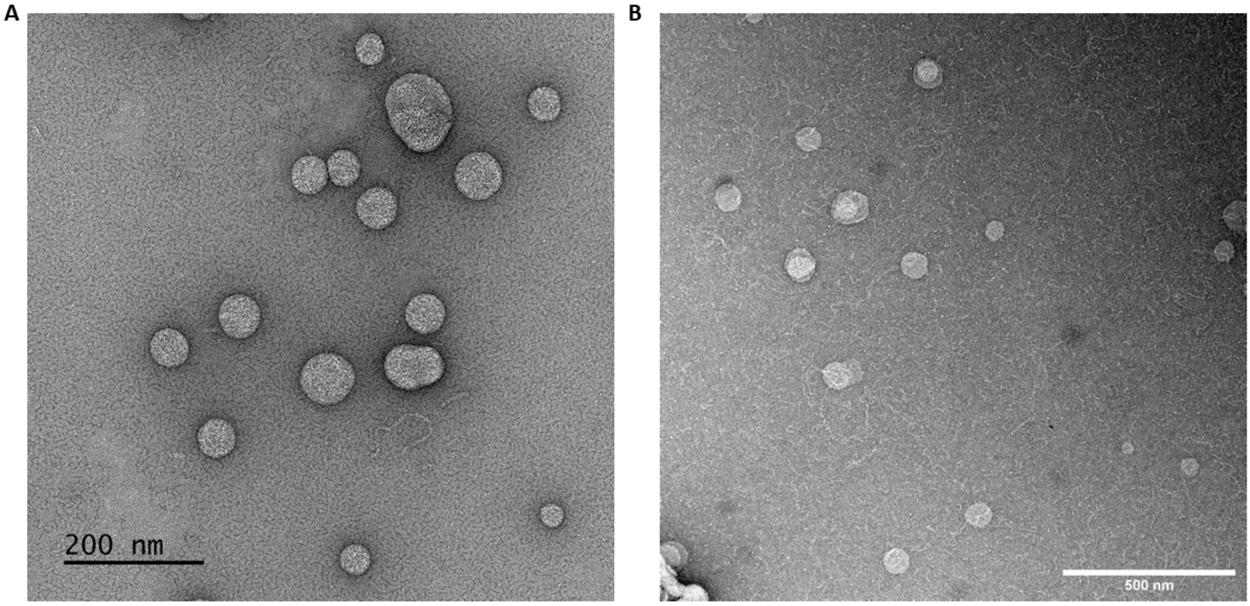

Figure S1. Comparison of lipofectamine 2000 (L2K) morphology by TEM performed using different grids. (A) L2K was deposited on a carbon-coated 400 mesh Cu/Rh grid and stained with 1% Uranyl Acetate from Polysciences, Warrington, PA, USA; the images were obtained using FEI Talos L120C TEM with Gatan 4k x 4k OneView camera. **(B)**L2K was deposited on a glow-discharged carbon-coated 200 mesh Cu grid and stained using 1% Uranyl Acetate from EMS, Hatfield, PA, USA; the images were obtained using FEI Tecnai T20 transmission electron microscope operating at 200 kV with a Gatan 2k X 2k Eagle camera. Other details of sample preparation were the same for image A and B as described in materials and methods.
